# Verification of the persistence of sperm traces under different chain-of-custody conditions. Care pathway and justice for victims of sexual violence and abuse

**DOI:** 10.1007/s12024-025-01149-7

**Published:** 2025-12-13

**Authors:** Antonina Argo, Maria Puntarello, Ginevra Malta, Martina Tarantino, Mauro Midiri, Simona Pellerito, Giuseppe Davide Albano, Stefania Zerbo

**Affiliations:** 1 Department of Health Promotion, Mother and Childcare, Internal Medicine and Medical Specialties (PROMISE), University of Palermo, Palermo, Italy; 2 Molecular and Health Biology, University of Palermo, Palermo, Italy

**Keywords:** Chain of custody, Sexual violence, Abuse, Forensic genetics

## Abstract

The primary aim of this study was to verify the persistence of seminal traces under varying chain-of-custody conditions, along with determining how different contamination factors, time intervals between collection, and storage methods influence the detectability of semen in the context of sexual assault cases. This study combined laboratory and field analyses to simulate real-case scenarios. Three forensic detection tools—Sperm Tracker Lab, Sperm Tracker Spray, and RSID™ tests—were evaluated on multiple substrates (skin, hair, nylon, cotton, and car interiors) and under various contamination conditions, including the presence of blood, dust, soil, and bodily fluids. Detection techniques included contact-pressure methods (Sperm Tracker Lab), application on uneven surfaces (Sperm Tracker Spray), fluorescence-based searches with ALS (alternative light sources), and immunochromatographic testing (RSID™ kits) for sperm-specific proteins. Positive findings were confirmed via microscopic examination and DNA analysis. All the samples were labelled and stored following strict chain-of-custody protocols. Sperm Tracker Spray demonstrated consistent effectiveness, successfully detecting minimal volumes (1–2 µL) across a wide range of materials. Conversely, ALS showed reduced sensitivity, especially in the presence of diluted or minimal traces and on textured or dark fabrics. RSID™ kits provided reliable confirmation of the presence of semen, even when environmental or biological contamination was present. Accurate and thorough documentation of the chain of custody proved essential for preserving sample authenticity and reducing the risk of error. The findings underscore the importance of a multidisciplinary forensic approach combining specialized reagents, confirmatory immunochromatographic testing, and rigorous adherence to chain-of-custody procedures. This integrated strategy enhances the reliability of seminal trace detection in investigations of sexual assault. Moreover, verifying trace persistence under diverse conditions contributes significantly to the evidentiary value of forensic samples in judicial contexts.

## Introduction

In accordance with the guidelines of Italian Forensic Geneticists (GE.F.I.), semen detection and the preservation of biological traces must follow standardized procedures to ensure analytical reliability and legal admissibility [1]. The chain of custody provides a systematic way to document each stage of evidence handling, from crime scene collection to laboratory analysis and eventual presentation in court [2]. In sexual assault cases, few forms of physical evidence are more critical than seminal evidence. When identified and genetically matched, semen can be a key piece of probative evidence [2, 3]. Detecting semen poses challenges, including suspect attempts to remove or obscure it and natural degradation from time or atmospheric exposure. Preserving these traces is paramount to securing valid, reliable forensic outcomes [3]. Without a trustworthy chain of custody, even the best forensic techniques can be undermined, risking loss or misinterpretation of crucial evidence [4, 5]. In investigations of sexual assault, detecting even very small amounts of seminal fluid is crucial [6]. Traditional screening often uses alternative light sources (ALS), which exploit the natural fluorescence of dried semen. However, ALS has limitations: Other bodily fluids, cosmetics, and cleaning agents can show similar fluorescence, causing false positives or false negatives. Microscopic identification of sperm cells is labour intensive, especially when sperm are rare or absent (vasectomy, oligospermia) [7, 8]. Morphological analysis can be further impeded by fragmented cells or conditions such as aspermia. Newer technologies include the Sperm Tracker line, available as a contact-pressure version (Sperm Tracker Lab^®^) for flat surfaces or an aerosol version (Sperm Tracker Spray^®^) for irregular or large surfaces (vehicles, skin). Both methods detect acid phosphatase, which is abundant in seminal fluid [9]. Immunochromatographic tests (RSID™) confirm semen by detecting semenogelins with fewer false positives than older acid phosphatase-only methods do [10]. The chain-of-custody process meticulously tracks each sample from collection through interpretation; gaps can make analyses inadmissible. This study aimed to evaluate the reliability of new detection tools in simulated scenarios and confirm a rigorous chain-of-custody practices, aligned with national guidelines, to safeguard seminal trace probative value. We examine integrated procedures that optimize forensic analysis in sexual assault cases. Combining strict ethical standards with advanced detection methods increases admissibility and decreases errors in sexual assault case outcomes [11–15]. Overall, these approaches can substantially improve investigative accuracy, offering faster detection, minimized contamination, and robust legal defensibility for forensic evidence [16, 17].

## Materials and methods

A study protocol detailing the experimental design, volunteer recruitment, and data management was submitted to the local Ethics Committee and approved. All the donors signed informed consent forms explaining the study’s purpose and ensuring the anonymity of the biological material, which was disposed of in accordance with regulations. To detect biological residues, several forensic tools were employed:

- A Sperm Tracker Lab^®^ (contact method) was applied to the suspicious fabrics after the reagent was moistened, and the samples were examined under UV light (365 nm). The blue luminescence indicates acid phosphatase activity.
- Sperm Tracker Spray^®^ (aerosol) was prepared by diluting the reagent in demineralized water and spraying it onto surfaces. The fluorescence was checked under UV light both immediately and after 5 min.
- Alternative light sources (LED 445 nm and a HandScope^®^ xenon lamp with filters) were used with orange goggles to classify the fluorescence as strong, weak, or absent.
- RSID™-Sperm kits confirmed positive results by detecting semenogelin, a semen-specific protein. Signal intensity was rated on a 0–5 scale.

Four scenarios were designed to test sperm detection under varying conditions, including contamination (dust, blood, and soil), washing, and delays of up to 72 h. Semen was deposited on selected surfaces and exposed to these variables. Chain-of-custody documentation was completed for each sample, including date, time, donor code, and storage conditions. The samples were transported in sealed, tamper-evident containers and refrigerated at 4 °C when not immediately processed. Removable items (e.g., fabric and seat covers) were bagged and coded. For nonremovable surfaces, such as skin or vehicle seats, Sperm Tracker Spray^®^ was applied onsite to reduce handling. Specifically:

- In the first scenario, which simulated an assault by multiple perpetrators, semen was applied to the skin (legs, chest, and arm) and hair to assess STK spray detection. The areas were cleaned, dried, and treated with 100 µL, 500 µL, or 1 mL of semen. After drying (30 min for skin, ~ 1 h for hair), STK Spray was applied. To assess interference, skin and hair treated with 100 µL of semen were contaminated with saliva, blood, or vaginal secretions. After 30 min of drying, RSID™ Sperm was applied per the manufacturer’s instructions. Positive results showed a red test line; controls with contaminants only were negative.
- The second scenario examined a sexual assault with vaginal penetration while the victim was clothed, leaving possible seminal traces inside the vaginal canal and on external garments. We tested Sperm Tracker^®^ (STK Lab device) on cotton (jeans) and nylon (underwear) fabric squares (10 × 10 cm). Each sample was cleaned with isopropyl alcohol and air-dried for 24 h to eliminate potential residues. A standardized amount of 50 µL of semen was then pipetted onto each square and dried for 30 min under controlled conditions. In accordance with the manufacturer’s guidelines, the STK Lab device was used to detect seminal fluid in the treated samples. To explore environmental contamination, some samples were left unsealed for 48 h, and dust was collected before RSID™ Sperm testing. Positive controls (semen-only) validated the test’s sensitivity, whereas negative controls (dust-only) showed no false positives.
- The third scenario simulated a vehicle-based sexual assault scenario in which semen may remain on car seats, seatbelts, and floor mats. All surfaces were first cleaned of debris, and then 50 µL of semen was applied to each 10 × 10 cm area and dried under controlled conditions. The Sperm Tracker STK Lab, calibrated with negative controls, was used to detect seminal fluid on car seats and seatbelts under normal and artificial lighting. Floor mats were tested using STK Spray. Soil contamination was simulated by uniformly applying 1 gram of agricultural soil over each semen-treated area. After one hour of drying at room temperature, RSID™ Sperm was used; positive results appeared where semen was present, with no false positives on soil-only controls. UV inspections and microscopic checks corroborated these findings.
- The fourth scenario addressed a male-on-male sexual assault, where the perpetrator allegedly forced oral acts. Trace semen may be deposited on the mouth, surrounding skin, or other exposed areas. To investigate the detection of different skin types, volunteers (or artificial surfaces) were deliberately contaminated with 100 µL of semen on the face, hand, and neck. Each region was first wiped with a sterile cloth, and after semen application, it was allowed to dry. Sperm Spray (STR Spray) was then nebulized over the treated zones. A visible blue colour developed within minutes, indicating the presence of sperm antigens. To assess how storage might affect detection, the samples were kept at room temperature (approximately 25 °C) for 24, 48, or 72 h. After each interval, a portion of the dried semen was mixed in RSID™ Sperm buffer and applied to the test cassette.

## Results

**Fig. 1:**
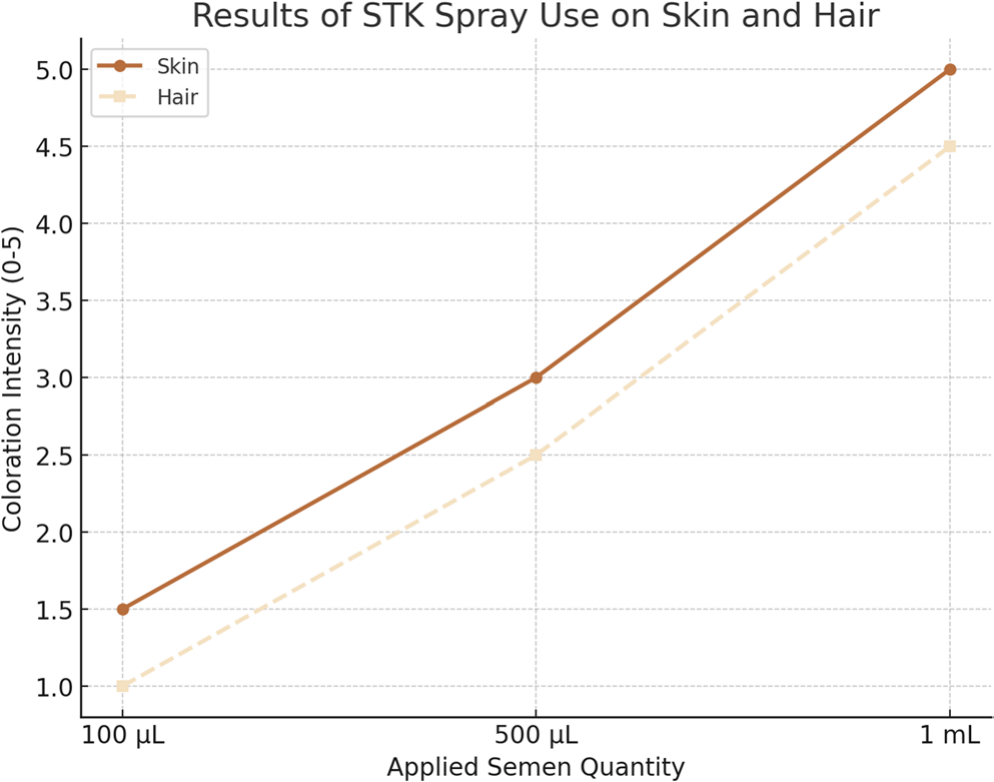
Fluorescence intensity detected on skin and hair after the application of various amounts of semen, following the use of STK Spray

**Fig. 2:**
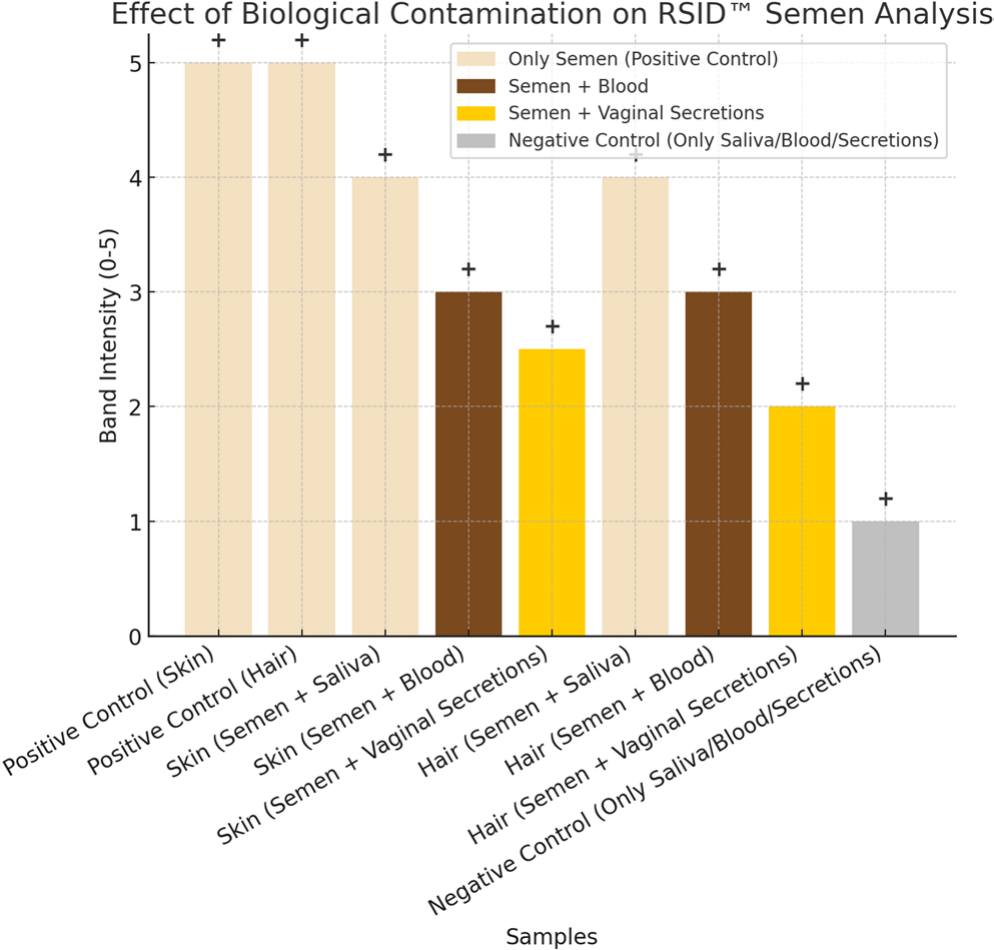
Intensity of positive bands (including controls) of the RSID-semen test after the application of seminal fluid and biological contaminants on areas of skin and hair

In the first scenario, a blue colour indicating sperm antigens appeared within 5–10 min. On the skin, 100 µL yielded a faint but visible reaction, increasing in intensity with increasing volume. On hair, 100 µL produced weak signals requiring close inspection, whereas 500 µL and 1 mL yielded progressively clearer results, with the latter visible to the naked eye (Fig. 1). Despite the added substances, RSID™ consistently confirmed the presence of semen. Saliva caused minimal interference, yielding clear test lines on both the skin and the hair. Blood and vaginal secretions slightly reduced the line intensity, especially on the skin, but did not prevent detection. STK Spray enabled rapid on-site screening, whereas RSID™ Sperm ensured reliable confirmation even under complex conditions (Fig. 2).

**Fig. 3:**
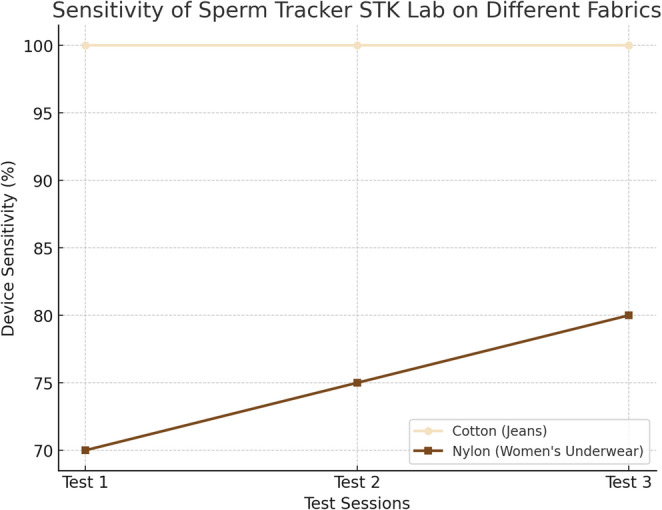
Sensitivity of the STK Tracker device in detecting seminal traces on fabrics such as cotton and nylon in each of the repetitions

**Fig. 4:**
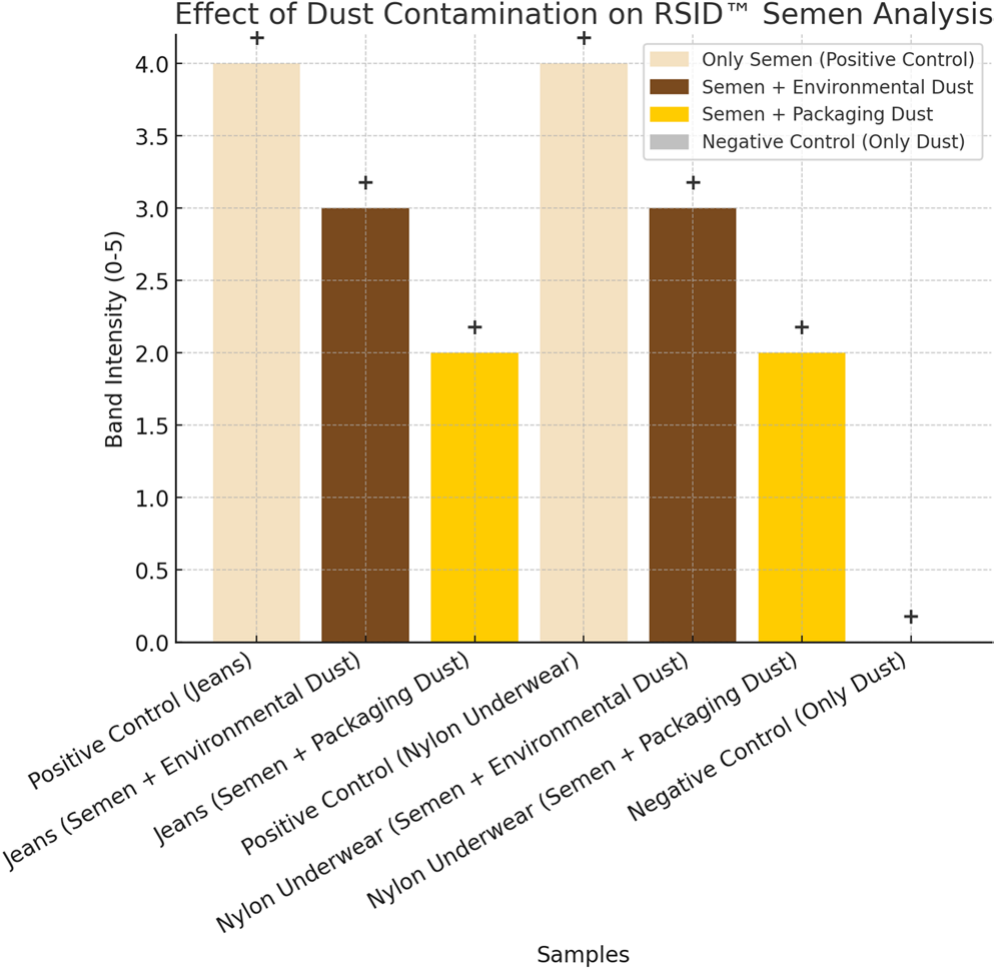
Intensity of the positive band (including controls) of the RSID-Semen tests after the application of seminal fluid and environmental contaminants (dust particles) on fabrics of different types

In the second scenario, the cotton squares showed 100% detection, with a strong link between the semen volume and the device response. In contrast, the nylon readings were more variable, likely because its synthetic fibres have lower permeability (Fig. 3). Applying light pressure improved the detection rates of nylon, reflecting how real-world wear might facilitate contact between semen and fabric fibres. Despite dust exposure, RSID™ Sperm consistently detected semen. Thus, the porous nature of cotton enhances the test results, but the use of nylon requires extra care or pressure. In both cases, combining Sperm Tracker and RSID™ Sperm offers reliable evidence of seminal traces on common garments. Jeans treated with semen alone yielded a strong test line (band intensity 4/5). Ambient dust contamination decreased the intensity to 3/5, and packaging dust further reduced it to 2/5. Women’s underwear produced comparable results (4/5, 3/5, 2/5). Although the line grew fainter with more dust, there were no false negatives, demonstrating RSID™ Sperm’s resilience against particulate contaminants (Fig. 4).

**Fig. 5:**
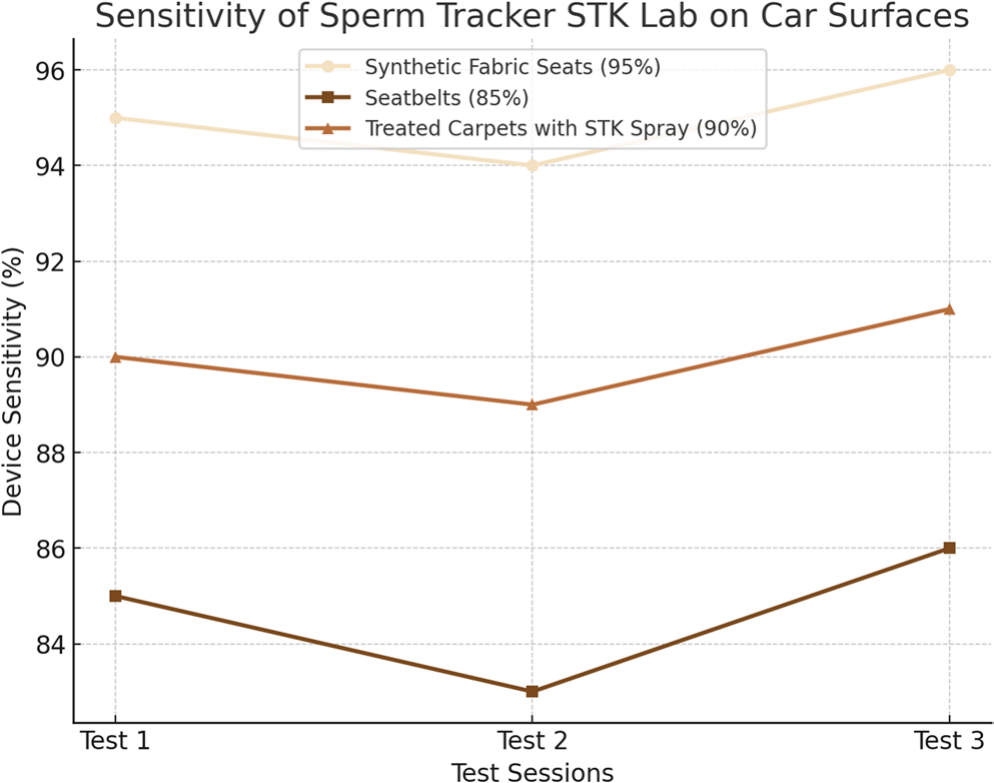
Sensitivity of the STK Tracker device in detecting seminal traces on surfaces of different types, such as sponges, polyesters, and fibrous material, in each of the repetitions performed

**Fig. 6:**
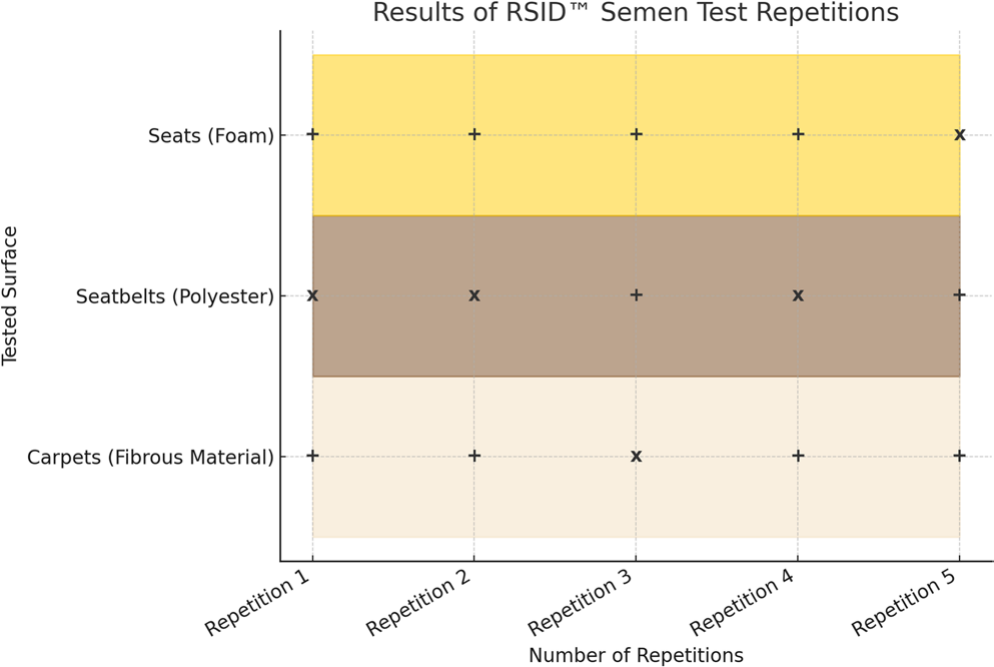
RSID test results in each repetition after the application of seminal fluid and an environmental contaminant (soil) on surfaces of different types

In the third scenario, approximately 95% sensitivity was achieved on synthetic seat fabric, with a slightly lower rate (85%) on seatbelts. This difference could be due to seatbelt materials having smoother surfaces, reducing fluid adherence and colour intensity. The STK Spray nebulized product was applied to the dried semen areas, after which the colour changes were observed after 30 min. Under challenging lighting conditions, STK Spray had a 90% detection rate. Overall, both the Sperm Tracker and STK Spray effectively identified semen on car seats, seatbelts, and floor mats, even with soil contamination, demonstrating strong forensic value in vehicle assault cases. Soil-contaminated synthetic car seat covers exhibited 95% sensitivity, with 4 of 5 repeats producing a positive result; the single negative result likely stemmed from uneven semen distribution. Polyester seatbelts, however, showed lower sensitivity (2 of 5 positives) and returned false negatives in 3 trials, reflecting how smoother surfaces hinder fluid retention. Carpeted floor mats scored 3 of 5 positives, with two false negatives linked to soil interference. While detection remained generally reliable, soil contamination increased the risk of nonuniform results (Figs. 5 and 6).

**Fig. 7:**
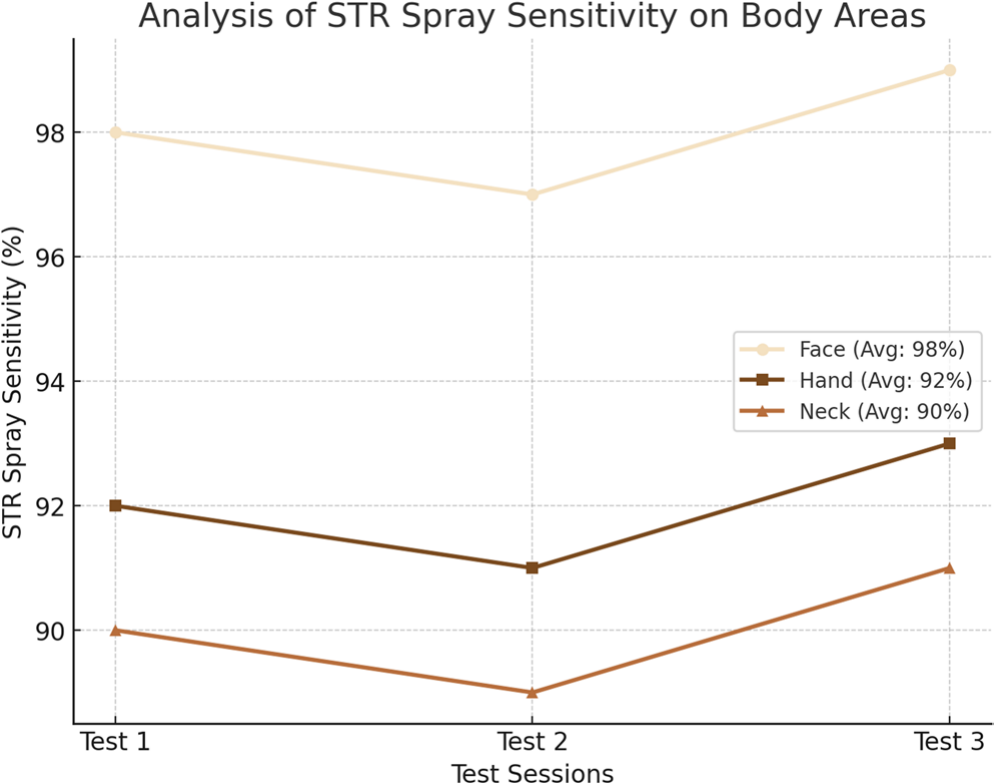
Sensitivity of the STK Spray device in detecting seminal traces on areas of skin (face, hand, and neck) in each of the repetitions performed

**Fig. 8:**
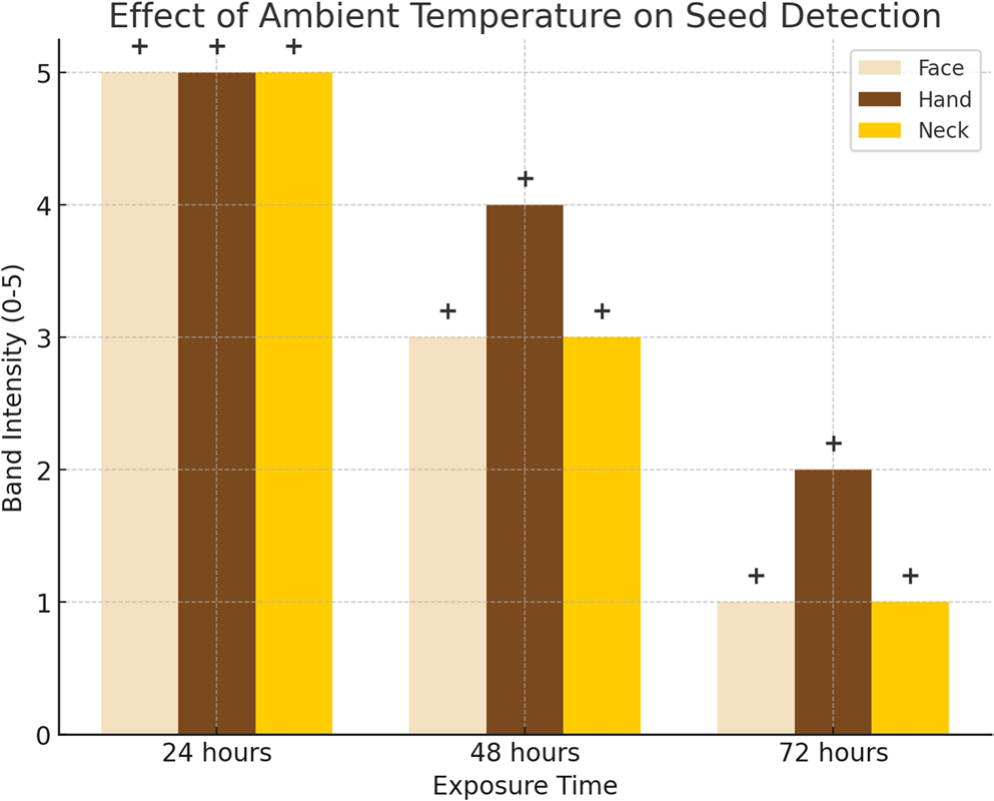
Intensity of the positive band of the RSID tests on skin areas (hand, face, and neck) containing seminal fluid exposed to suboptimal temperatures for periods of 24, 48, and 72 h

In the fourth scenario, the face, with higher sebum production, showed a quicker and more intense reaction, whereas the hand and neck were clearly positive, although with slightly reduced colour intensity. STR Spray also demonstrated high sensitivity for very small semen volumes (1–2 µL) (Fig. 7). After ambient contamination within 10–15 min, a red test line confirmed the presence of sperm-associated protein. These results underscore the effectiveness of STR Spray for detecting semen on male victims’ skin, including areas involved in oral sexual assault, and highlight the stability of seminal proteins over several days. Male-on-male assault samples stored at 25 °C for varying durations showed decreasing test-line intensity over time. At 24 h, the face, hand, and neck all tested strongly positive. By 48 h, the lines weakened slightly but were still clearly positive. At 72 h, detection decreased markedly, with the face and neck often showing faint or borderline lines; hand samples remained faintly positive. Thus, beyond 48 h at room temperature, the test reliability decreases significantly (Fig. 8).

Overall, RSID™ Sperm effectively detected semen on skin, hair, fabrics, and car materials, even with contaminants such as saliva, blood, vaginal secretions, dust, or soil. Although the intensity can decrease under adverse conditions, actual false negatives were uncommon, especially when surfaces allowed better fluid absorption. Prolonged storage at room temperature further diminished line visibility, emphasizing the importance of timely collection and analysis. Investigators should also note that consistent sampling, thorough documentation, and immediate preservation help maintain trace integrity throughout the forensic process. Nonetheless, RSID™ Sperm remains a valuable tool for investigations of sexual assault, offering robust results that support reliable forensic conclusions.

## Discussion

The multiscenario experimental design of this study offers insight into how environmental and biological variables influence the reliability of semen detection tools. Rather than drawing definitive conclusions, the data suggest patterns that warrant further investigation. The layered methodological approach—ALS screening followed by targeted AP‑based Sperm Tracker techniques and RSID™ confirmation—appears to reduce false negatives and improve the localization of semen traces [18–22]. However, the performance of ALS is variable, with fluorescence frequently affected by dyes, cosmetics, and fluids, an issue widely noted in the literature and deserving of more systematic study [23]. These findings further indicate that complex contaminants such as blood and faecal matter can weaken fluorescence or colorimetric reactions [24], raising questions about threshold sensitivity in real forensic settings where multiple victims or contributors may be present [25, 26]. Although RSID™ demonstrated strong robustness in this study, the mechanisms underlying its resilience in mixed-fluid environments should be explored in greater depth, particularly given the biochemical variability of body fluids [27]. Another element emerging from the results is the impact of environmental degradation on DNA recovery. Partial profiles were primarily linked to exposure conditions rather than interference from detection reagents [8, 28]. This underscores the importance of evaluating how different substrates, temperatures, and humidity levels alter both chemical and genetic trace stability. Systematic testing under controlled extreme environments may help refine best-practice protocols. Additional areas that merit further investigation include the performance of Sperm Tracker Spray^®^ on highly fibrous or high‑pile materials, the role of advanced lighting systems or imaging analysis in reducing operator‑dependent variability, and the potential for cross‑reactivity in complex biological mixtures with possible contribution from several individuals [29, 30]. Expanding research into new biomarkers—particularly for cases involving aspermia or vasectomy—may also broaden the applicability of immunochromatographic testing. Overall, the study highlights several strengths of the current detection workflow but also reveals domains where empirical data remain limited. These observations reinforce the need for continued experimentation to determine detection thresholds, substrate‑specific behaviours, and environmental impacts on both chemical and genetic evidence integrity [31, 32]. A more extensive dataset could clarify how technology–protocol integration influences investigative efficiency and evidentiary reliability in sexual assault cases [33, 34].

## Conclusions

This study highlights how integrating advanced detection reagents (Sperm Tracker Spray, Sperm Tracker Lab) with confirmatory tests (RSID™) substantially strengthens forensic investigations in sexual assault cases. Owing to rigorous sample preservation under chain-of-custody procedures and the use of specialized detection methods, forensic teams can identify and confirm even minimal or partially degraded semen traces on difficult surfaces. Key findings include the following:

- High Sensitivity: Sperm Tracker Spray detects very small volumes (1–2 µL) of a variety of absorbent and nonabsorbent materials, maintaining efficacy even after mild washing or contamination attempts.
- Adaptability to Various Scenarios: The contact (Lab) and aerosol (Spray) methods complement each other, enabling the analysis of both removable fabrics and fixed or curved surfaces.
- Essential Chain of Custody: Labelling, documentation, and secure storage ensure that evidence is admissible in court and preserve scientific integrity.
- Resistance to Contaminants: Although blood and faecal matter can impede or weaken fluorescent signals, a second application of Spray and RSID^™^ confirmation often reveals hidden semen.
- Minimal Impact on DNA: The use of Sperm Tracker had no significant negative effect on subsequent STR analysis for suspect identification.

On the basis of these findings, a “layered” approach is recommended: initial rapid screening with ALS, targeted use of Sperm Tracker (Spray or Lab) in suspicious areas, confirmation with RSID™ kits, and possible microscopic or genetic analysis if specific suspect identification is needed [11, 36–39]. The synergy of these methods, supported by strict adherence to chain-of-custody protocols, enables the collection of scientifically solid and reliable evidence in sexual assault cases [11, 37–40]. Future studies may expand on these conclusions by testing additional surfaces (synthetic leathers, painted walls, and high-pile carpets), exploring advanced digital image analysis techniques, and developing new chemical markers for vasectomy or aspermia cases [44, 45]. These perspectives align with recent research exploring alternative molecular markers to enhance fluid differentiation, supporting the continuous refinement of forensic identification strategies [46]. Nevertheless, the evidence presented here indicates a significant improvement in detection rates and probative reliability over older screening methods. Unconditional adherence to chain-of-custody protocols and the use of high-sensitivity testing constitute a decisive combination for successful forensic outcomes.

## Key points

1. Spermatozoa detection is consistently maintained across the simulated custodial degradation scenarios.
2. GE.F.I.- standardized protocols optimize biomolecular trace integrity and forensic yield.
3. Integration of forensic procedures within multidisciplinary sexual assault pathways reinforces evidentiary robustness.
4. The results substantiate the medico-legal reliability of seminal fluid analysis for judicial admissibility.

## References

[1] G. Genetisti Forensi Italiani Presidente, F. De Stefano, P. Susi Pelotti, D. Loredana Buscemi, D. Eugenia Carnevali, And P. Francesco De Stefano, “Genetisti Forensi Italiani Associazione Scientifica Genetisti Forensi Italiani (Ge.F.I).”

[2] D’Anna T, Puntarello M, Cannella G, et al. The Chain of Custody in the Era of Modern Forensics: From the Classic Procedures for Gathering Evidence to the New Challenges Related to Digital Data. Healthcare (Basel). 2023;11(5):634. Published 2023 Feb 21. doi:10.3390/healthcare11050634 10.3390/healthcare11050634 PMC10000967 36900637

[3] Wood GJ, Smith JAS, Gall JA. The optimal timing of forensic evidence collection following paediatric sexual assault. J Forensic Leg Med. 2023;95:102499. 10.1016/j.jflm.2023.102499. 36889049 10.1016/j.jflm.2023.102499

[4] Rasool N, Rasool M. DNA evidence in sexual assault cases in Pakistan. Med Sci Law. 2020;60(4):270–7. 10.1177/0025802420934240. 32576088 10.1177/0025802420934240

[5] Kampmann ML, Tfelt-Hansen J, Børsting C. Cleaning protocols in forensic genetic laboratories. Int J Legal Med. 2024;138(5):1787–90. 10.1007/s00414-024-03232-0. 38649547 10.1007/s00414-024-03232-0 PMC11306349

[6] Kane D, Walshe J, Richardson D, et al. Storage of evidence and delayed reporting after sexual assault: rates and impact factors on subsequent reporting. J Forensic Leg Med. 2024;106:102731. 10.1016/j.jflm.2024.102731. 39128277 10.1016/j.jflm.2024.102731

[7] Sato I, Kojima K, Yamasaki T, et al. Rapid detection of Semenogelin by one-step immunochromatographic assay for semen identification. J Immunol Methods. 2004;287(1–2):137–45. 10.1016/J.Jim.2004.01.017. 15099762 10.1016/j.jim.2004.01.017

[8] Martínez P, Santiago B, Alcalá B, Atienza I. Semen searching when sperm is absent. Sci Justice. 2015;55(2):118–23. 10.1016/j.scijus.2015.01.008. 25753997 10.1016/j.scijus.2015.01.008

[9] Bernabe M. S., “Performance Characteristics Of Sperm Detection By Microscopy And Purple Color Test For Acid Phosphatase In Vaginal Swabs Of Sexual Crime Survivors.” PUP Journal of Science and Technology. Dec 2021. DOI: 10.70922/mewg8e16

[10] Morrison J, Watts G, Hobbs G, Elsevier Ireland Ltd, et al. Field-based detection of biological samples for forensic analysis: established techniques, novel tools, and future innovations. Forensic Sci Int. 2018. 10.1016/J.Forsciint.2018.02.002. 10.1016/j.forsciint.2018.02.002 29518713

[11] Malta G, Puntarello M, Midiri M, D’Anna T, Zerbo S, Argo A. Forensic homicidal strangulation in women: case series and systematic literature review. Forensic Sci Int Synerg. 2025;10:100577. 10.1016/j.fsisyn.2025.100577. 40034148 10.1016/j.fsisyn.2025.100577 PMC11875827

[12] Kaur S, Rawat B, Springer Science And Business Media Deutschland Gmbh. Medico-legal evidence collection in child sexual assault cases: a forensic significance. Egypt J Forensic Sci. 2021(1). 10.1186/S41935-021-00258-Y.

[13] Kidenda S, Muchai R, Green L, McHale T, Mishori R, Nelson BD. Evaluating the effectiveness of a mobile application to improve the quality, collection, and usability of forensic documentation of sexual violence. PLoS One. 2022;17(12):e0278312. 10.1371/journal.pone.0278312. 36516163 10.1371/journal.pone.0278312 PMC9750009

[14] Wohlfahrt D M, “Developmental Validation Of A Bacterial Signature-Based Developmental Validation Of A Bacterial Signature-Based Identification Method Of Forensically Relevant Human Biological Identification Method Of Forensically Relevant Human Biological Samples Samples.” [Online]. Available: Https://Scholarscompass.Vcu.Edu/Etd

[15] Woollacott C, Goray M, Van Oorschot RAH, et al. The transfer, prevalence, persistence, and recovery of DNA from body areas in forensic science: a review. Forensic Sci. 2025;5(1):9. 10.3390/Forensicsci5010009.

[16] Sijen T, Harbison S. On the identification of body fluids and tissues: a crucial link in the investigation and solution of crime. Genes. 2021;12(11):1728. 10.3390/genes12111728. 34828334 10.3390/genes12111728 PMC8617621

[17] Gino et al. 58 cases of sexual violence bearing forensic interest: congruence between the victim’s report and the data from laboratory analyses. Int J Legal Med. 2017;131(5):1449–53. 10.1007/S00414-017-1602-X. 10.1007/s00414-017-1602-x 28488000

[18] Abdel Rahman A, Khater S, Attia E. Rapid stain identification (Rsid-Tmsemen): a rapid tool for seminal fluid detection. Ain Shams Journal Of Forensic Medicine And Clinical Toxicology. 2020;35(2):73–80. 10.21608/Ajfm.2020.111862.

[19] van Oorschot RAH, Meakin GE, Kokshoorn B, Goray M, Szkuta B. DNA transfer in forensic science: recent progress towards meeting challenges. Genes. 2021;12(11):1766. 10.3390/genes12111766. 34828372 10.3390/genes12111766 PMC8618004

[20] Kim JY, Kim MI, Lee HH, et al. Application of hematoxylin reagent for sperm cell separation in sexual crime evidence. Forensic Sci Int. 2020;307:110114. 10.1016/j.forsciint.2019.110114. 31901461 10.1016/j.forsciint.2019.110114

[21] Fejes V, Simon G, Makszin L, Sipos K, Poor VS. Evaluation of the effect of ozone disinfection on forensic identification of blood, saliva, and semen stains. Sci Justice. 2024;64(2):151–8. 10.1016/j.scijus.2023.12.005. 38431372 10.1016/j.scijus.2023.12.005

[22] Old J, Schweers BA, Boonlayangoor PW, Fischer B, Miller KW, Reich K. Developmental validation of RSID™-semen: a lateral flow immunochromatographic strip test for the forensic detection of human semen. J Forensic Sci. 2012;57(2):489–99. 10.1111/j.1556-4029.2011.01968.x. 22211796 10.1111/j.1556-4029.2011.01968.x

[23] Evers H, Heidorn F, Gruber C, Lasczkowski G, Riße M, Dettmeyer R, et al. Investigative strategy for the forensic detection of sperm traces. Forensic Sci Med Pathol. 2009;5:182–8. 10.1007/s12024-009-9092-x. 19517276 10.1007/s12024-009-9092-x

[24] Yao T, Han X, Guan T, et al. Effect of indoor environmental exposure on seminal microbiota and its application in body fluid identification. Forensic Sci Int. 2020;314:110417. 10.1016/j.forsciint.2020.110417. 32702532 10.1016/j.forsciint.2020.110417

[25] Lincoln CA. Sexual assault: forensic examination in the living and deceased. Acad Forensic Pathol. 2018;8(4):912–23. 10.1177/1925362118821490. 31240080 10.1177/1925362118821490 PMC6491537

[26] Pradeep AS, Babu J, Sudaroli Sandana J, Deivalakshmi S. Innovations in forensic science: comprehensive review of hyperspectral imaging for bodily fluid analysis. Forensic Sci Int. 2024;364:112227. 10.1016/j.forsciint.2024.112227. 39278154 10.1016/j.forsciint.2024.112227

[27] Smith DA, Webb LG, Fennell AI, Nathan EA, Bassindale CA, Phillips MA. Early evidence kits in sexual assault: an observational study of spermatozoa detection in urine and other forensic specimens. Forensic Sci Med Pathol. 2014;10:336–43. 10.1007/s12024-014-9562-7. 24752424 10.1007/s12024-014-9562-7

[28] Habek D, Habek D. Women’s sexual abuse: forensic-gynecologic expertise experiences. Forensic Sci Med Pathol. 2023;19:617–9. 10.1007/s12024-023-00626-1. 37148439 10.1007/s12024-023-00626-1

[29] Kamodyová N, Durdiankovà J, Celec P et al“Prevalence And Persistence Of Male Dna Identified In Mixed Saliva Samples After Intense Kissing,” Forensic Sci Int Genet, Vol. 7, No. 1, Pp. 124–128, Jan. 2013, Doi: 10.1016/J.Fsigen.2012.07.007. 10.1016/j.fsigen.2012.07.007 22917815

[30] Swayambhu M, Gysi M, Haas C, et al. Standardizing a microbiome pipeline for body fluid identification from complex crime scene stains. Appl Environ Microbiol. 2025;91(5):e0187124. doi:10.1128/aem.01871-24 10.1128/aem.01871-24 PMC12093949 40304519

[31] Tullio V, Lanzarone A, Scalici E, Vella M, Argo A, Zerbo S. Violence against women in heterosexual couples: a review of psychological and medico-legal considerations. Med Sci Law. 2021;61(1_suppl):113–24. 10.1177/0025802420936081. 33591871 10.1177/0025802420936081

[32] Holtkötter H, Schwender K, Wiegand P, Peiffer H, Vennemann M. Improving body fluid identification in forensic trace evidence-construction of an immunochromatographic test array to rapidly detect up to five body fluids simultaneously. Int J Legal Med. 2018;132(1):83–90. 10.1007/s00414-017-1724-1. 29082429 10.1007/s00414-017-1724-1

[33] Menaker TA, Campbell BA, Wells W. The use of forensic evidence in sexual assault investigations: perceptions of sex crimes investigators. Violence Against Women. 2017;23(4):399–425. 10.1177/1077801216641519. 27094435 10.1177/1077801216641519

[34] Chalmers K, Hollender M, Spurr L, et al. Emergency department preparedness to care for sexual assault survivors: a nationwide study. West J Emerg Med. 2023;24(3):629–36. 10.5811/westjem.59257. 37278801 10.5811/westjem.59257 PMC10284505

[35] Williams CM. Evidentiary discrepancies in sexual assault casework within the US. Forensic Sci Res. 2021;6(3):189–94. 10.1080/20961790.2021.1960465. 34868710 10.1080/20961790.2021.1960465 PMC8635549

[36] Dash HR. Advancements in differentiation between sperm cells and epithelial cells for efficient forensic DNA analysis in sexual assault cases. Int J Legal Med. 2024;138(6):2209–27. 10.1007/s00414-024-03285-1. 38995400 10.1007/s00414-024-03285-1

[37] Johnson D, Peterson J, Sommers I, Baskin D. Use of forensic science in investigating crimes of sexual violence: contrasting its theoretical potential with empirical realities. Violence Against Women. 2012;18(2):193–222. 10.1177/1077801212440157. 22433228 10.1177/1077801212440157

[38] Watanabe K, Akutsu T, Sakurada K. Development of a Real-Time PCR-Based Method for Analyzing Semen-Specific Unmethylated DNA Regions and Methylation Status in Aged Body Fluid Stains. J Forensic Sci. 2016;61(1):S208–12. 10.1111/1556-4029.12941. 26305854 10.1111/1556-4029.12941

[39] Xiao Y, Chen D, Peng D, et al. Establishment of a co-analysis system for personal identification and body fluid identification: a preliminary report. Int J Legal Med. 2022;136(6):1565–75. 10.1007/s00414-022-02886-y. 36076078 10.1007/s00414-022-02886-y

[40] Ribéreau-Gayon A, Rando C, Schuliar Y, et al. Extensive unusual lesions on a large number of immersed human victims found to be from cookiecutter sharks (*Isistius* spp.): an examination of the Yemenia plane crash. Int J Legal Med. 2017;131(2):423–32. 10.1007/s00414-016-1449-6. 27623973 10.1007/s00414-016-1449-6 PMC5306341

[41] Magalhães T, Dinis-Oliveira RJ, Silva B, Corte-Real F, Nuno Vieira D. Biological evidence management for DNA analysis in cases of sexual assault. Sci World J. 2015;2015:365674. 10.1155/2015/365674. 10.1155/2015/365674 PMC4637504 26587562

[42] Basset P, Blandin P, Grini A, Delemont S, Samie L, Castella V. A simplified protocol for the detection of blood, saliva, and semen from a single biological trace using immunochromatographic tests. Forensic Sci Med Pathol. 2022;18(2):141–8. 10.1007/s12024-021-00453-2. 35171453 10.1007/s12024-021-00453-2 PMC9106612

[43] Gooch J, Tungsirisurp S, Costanzo H, Napier R, Frascione N. Generating aptamers towards human sperm cells using massively parallel sequencing. Anal Bioanal Chem. 2021;413(23):5821–34. 10.1007/s00216-021-03562-7. 34355252 10.1007/s00216-021-03562-7 PMC8437879

[44] Zhao M, Cai M, Lei F, et al. AI-driven feature selection and epigenetic pattern analysis: a screening strategy of CpGs validated by pyrosequencing for body fluid identification. Forensic Sci Int. 2025;367:112339. 10.1016/j.forsciint.2024.112339. 39729807 10.1016/j.forsciint.2024.112339

[45] Murphy C, Alexander K, Stark MM, Davidson G. Novel recovery methods for biological materials in cases of alleged sexual assault: a word of caution. Sci Justice. 2022;62(5):621–3. 10.1016/j.scijus.2022.09.005. 36336455 10.1016/j.scijus.2022.09.005

[46] Alsaeed SA, Elrewieny NM, Eltokhy RAA, Mohamed MS, Khalil WKB, Shalby AB, et al. Analysis of MiR-20b and miR-197 markers for differentiation between forensic body fluids encountered in sexual assault cases. Forensic Sci Med Pathol. 2025;21:56–62. 10.1007/s12024-024-00831-6. 38856935 10.1007/s12024-024-00831-6 PMC11953183

